# A global mathematical model of climatic suitability for *Plasmodium falciparum* malaria

**DOI:** 10.1186/s12936-024-05122-7

**Published:** 2024-10-10

**Authors:** Owen Brown, Jennifer A. Flegg, Daniel J. Weiss, Nick Golding

**Affiliations:** 1 School of Mathematics and Statistics, University of Melbourne, Melbourne, Australia; 2 The Kids Research Institute Australia, Perth Children’s Hospital, Nedlands, Australia; 3 School of Population Health, Curtin University, Bentley, Australia; 4 Melbourne School of Population and Global Health, University of Melbourne, Melbourne, Australia

**Keywords:** Malaria risk, Mathematical modelling, Computational model

## Abstract

Climatic conditions are a key determinant of malaria transmission intensity, through their impacts on both the parasite and its mosquito vectors. Mathematical models relating climatic conditions to malaria transmission can be used to develop spatial maps of climatic suitability for malaria. These maps underpin efforts to quantify the distribution and burden of malaria in humans, enabling improved monitoring and control. Previous work has developed mathematical models and global maps for the suitability of temperature for malaria transmission. In this paper, existing temperature-based models are extended to include two other important bioclimatic factors: humidity and rainfall. This model is combined with fine spatial resolution climatic data to produce a more biologically-realistic global map of climatic suitability for *Plasmodium falciparum* malaria. The climatic suitability index developed corresponds more closely than previous temperature suitability indices with the global distribution of *P. falciparum* malaria. There is weak agreement between the Malaria Atlas Project estimates of *P. falciparum* prevalence in Africa and the estimates of suitability solely based on temperature (Spearman Correlation coefficient of $\rho = 0.24$). The addition of humidity and then rainfall improves the comparison ($\rho = 0.62$ when humidity added; $\rho =0.70$ when both humidity and rainfall added). By incorporating the impacts of humidity and rainfall, this model identifies arid regions that are not climatically suitable for transmission of *P. falciparum* malaria. Incorporation of this improved index of climatic suitability into geospatial models can improve global estimates of malaria prevalence and transmission intensity.

## Introduction

*Plasmodium falciparum* malaria poses an enormous health burden globally, with more than 200 million cases and 500,000 deaths per year. This burden is predominantly focussed in sub-Saharan Africa, where 90% of the continent’s population resides in *P. falciparum* malaria endemic areas [1]. The global impact of *P. falciparum* malaria has declined however, with unprecedented funding of both health systems and targetted malaria interventions (such as long-lasting insecticidal bed nets) resulting in a 30% reduction in cases from 2005 to 2015 [1–3]. Due to varying environmental conditions and spatially heterogeneous deployment and utilization of interventions, the intensity of malaria transmission, and hence the incidence of disease, varies substantially throughout endemic areas [4, 5]. High-resolution maps of malaria prevalence and incidence are therefore critical for measuring progress in tackling malaria. These maps can also be powerful tools for advocacy, targetting interventions at locations where they can have the greatest impact, and as early warning systems to support health systems to deal with malaria outbreaks [6].

Spatial variation in malaria transmission intensity is driven by myriad factors, including: climate, land cover, socioeconomic conditions, coverage of malaria interventions [7, 8], and growing resistance to them [3, 9, 10]. Geostatistical models can be used to combine spatial estimates of these drivers, along with data on malaria prevalence and incidence from health systems and surveys, into spatio-temporal surfaces of malaria prevalence and incidence [11–13]. As a correlative approach, geostatistical models do not explicitly capture the biological processes and causal relationships that link these risk factors to malaria transmission. This reduces their ability to identify the hard limits of disease transmission, and their reliance on spatial correlation to describe disease risk means they have limited ability to predict malaria transmission potential in areas far away from existing data points [14]. The limitations of a purely geostatistical approach, including those that use bioclimatic covariates in the statistical framework, to mapping global malaria transmission intensity can be overcome by incorporating predictions from causal mathematical models of the major processes driving spatial variation in malaria transmission [3, 12, 15, 16]. However, even with the inclusion of more appropriate bioclimatic variables in a mechanistic model to identify suitable habitats for malaria transmission, the ability to predict actual malaria transmission in these regions is fundamentally limited by access to reliable data on malaria interventions, antimalarial treatments etc.

Over the last few decades, several attempts have been made to mathematically model the relative risk of malaria in different locations, with each model using a variety of methods and taking into account different factors (a review of some of these models can be found in Reiner et al. [17]). These models can be applied to a variety of problems: knowing where malaria risk is highest allows treatment efforts and preventative measures to be applied in the areas where they are most needed. These models to predict how changes in local climate and environment may alter malaria risk. There is also a large body of work on dynamic mathematical models of malaria (see review by [18]) and agent-based modelling of malaria (reviewed by Smith et al. [19]).

Climatic suitability is critical requirement for malaria transmission, and a number of mathematical models have been developed to identify areas that are climatically suitable or unsuitable for disease transmission [15, 16, 20–22]. Attempts to generate global maps of climatic suitability for *P. falciparum* malaria have typically focussed on a single aspect: temperature. The effect of temperature on the breeding cycle of *Anopheles* vectors and *Plasmodium* sporozoites is well documented [23–25] and is the cause of *P. falciparum* malaria’s tropical distribution. Gething et al. [15] developed a global map of the temperature-based constraints on malaria transmission, using established mathematical models, and spatial data on average maximum and minimum air temperatures, and their seasonal variation. Weiss et al. [16] extended this work by applying the same model to high temporal- and spatial-resolution observed temperature data derived from satellite imagery. Whilst these studies provide detailed estimates of suitability for malaria based on the *direct* impacts of temperature, they ignore the crucial impact of precipitation on both the availability of water bodies for mosquito larval habitat, and on humidity, which has a major effect on mosquito survival. Consequently, the global suitability indices provided by these previous studies are unable to identify areas that are too dry for malaria, potentially overpredicting the climatic limits of transmission. Furthermore, this shortcoming necessitates the inclusion of variables that characterize moisture conditions in subsequent malaria burden models.

This paper outlines the first global-scale mathematical model that quantifies the direct, combined, impact of temperature, humidity and precipitation of malaria suitability. This enables climatic suitability mapping for malaria globally, and with greater biological realism than previous studies. The resulting map can be used in global geostatistical modelling studies to improve global estimates of malaria burden, or used to predict the likely impacts of changing climates on suitability for malaria.

## Methods and implementation

In this paper, a suitability index is developed for the transmission of *P. falciparum* that varies with temperature, humidity and rainfall. The modelling framework employs mathematical relationships between the environmental metrics and the *Anopheles* mosquito life-cycle. The model also considers the mechanistic relationship between temperature and the development of infectious sporozoites living within infected mosquitoes. The equations that characterize the relationships were previously established in peer-reviewed publications, however this is the first time the three climatic factors have been integrated into a single mathematical model. Critically, the input data for the models was produced using a well-established, geographically consistent methodology, thus ensuring comparability of the results through time and space. Challenges associated with this research include data management on a large set of global-scale gridded datasets and computational intensity of the analysis.

### Data

The mathematical model requires data on temperature, humidity and rainfall. All data was sourced from ERA5-Land via the Copernicus programme, taking data points at intervals every 2 h between March 2022 and May 2023 at a nominal 10 km resolution: pixels spanning 0.1 degrees of latitude/longitude [26].

Only the 2 m temperature variable was considered when measuring air temperature. Relative humidity was calculated by combining the air temperature data with the 2 m dewpoint temperature, and using the Magnus approximation:

1 $$\begin{aligned} M(x)&=\text {exp}\left( \frac{17.625x}{243.04 + x}\right) \end{aligned}$$

2 $$\begin{aligned} RH&=\frac{100M(D_{p})}{M(T)} \end{aligned}$$

where $D_{p}$ is the dewpoint temperature (°C), *T* is the air temperature (°C) and *RH* is the relative humidity [27].

The variables of total precipitation and evaporation from open water surfaces excluding oceans were also used in later models. Since the data provided in the dataset was cumulative over 24 h periods, the difference between data points was used to approximate the total precipitation and evaporation in a 2 h period.

### Temperature-dependent model

**Fig. 1:**
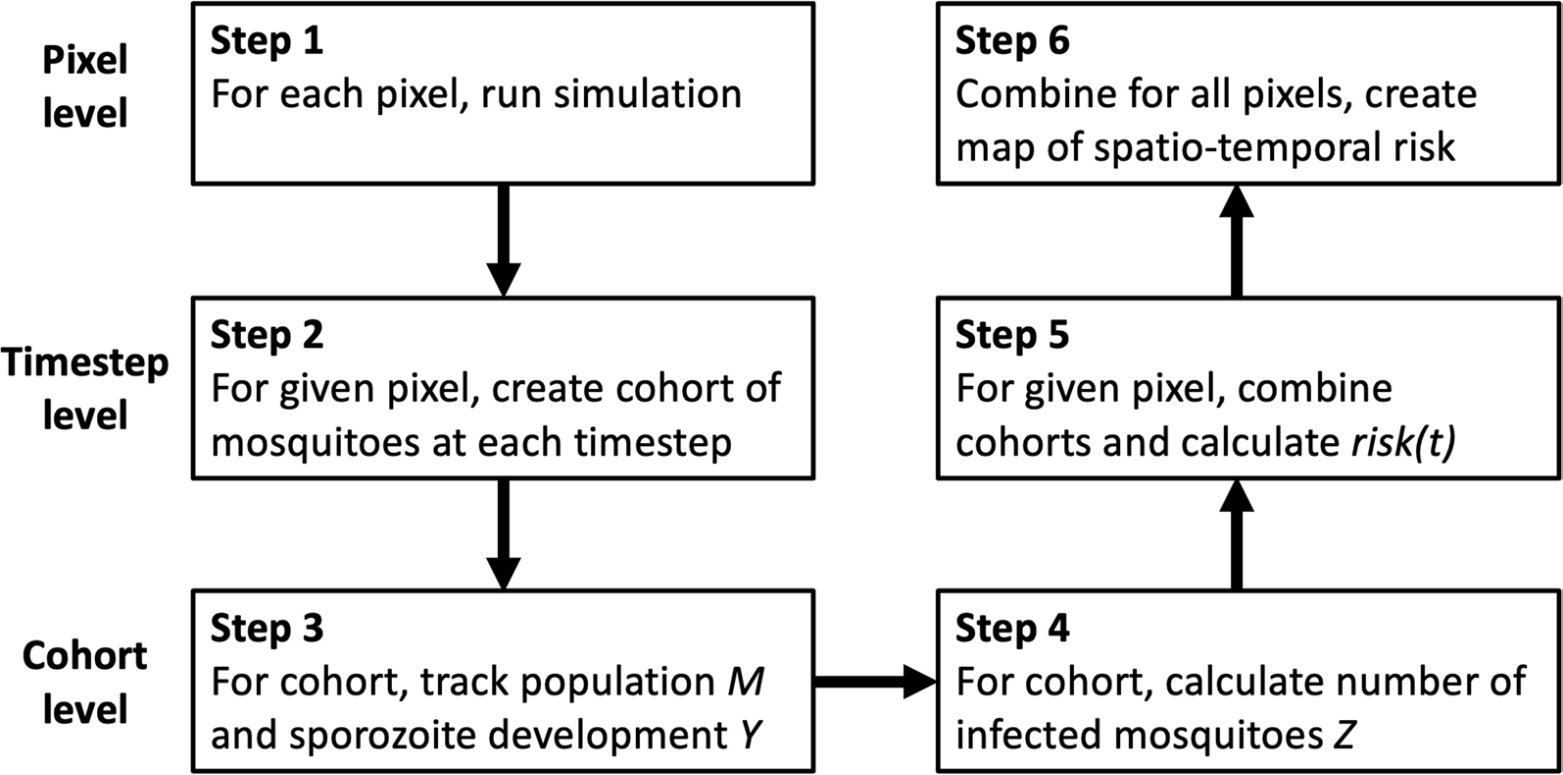
A flowchart describing the steps of initial temperature-based model. Starting with the raw inputs for each pixel of the map, many cohorts of mosquitoes are simulated, data is used to calculate the number of infected mosquitoes, this is then combined across all cohorts and pixels to produce a spatio-temporal map of malaria risk

A population model is formulated in a discrete-time framework to track mosquito infection and breeding dynamics, using techniques presented in Brady et al. [28]. See Fig. 1 for an overview of the model structure at the pixel (point location) level, time-step level and cohort level. At each pixel a separate simulation is run (Step 1, Fig. 1). For a given pixel, a new cohort of mosquitoes is created at each 2 h timestep, $t_i$ (Step 2, Fig. 1). For a given cohort, the population size and sporozoite development within the population is tracked at 2 hly intervals (Step 3, Fig. 1). To do this, $M_i$ is defined as the population of the cohort *i* timesteps ($t_i = 2i$ hours) after the adult phase of the mosquito life cycle commences and additionally:

3 $$\begin{aligned}&M_0=100,\end{aligned}$$

4 $$M_{{i + 1}} = \exp \left( {\frac{{ - g(T)}}{{12}}} \right)M_{i} ,\quad i = 0, \ldots ,395$$

where *g*(*T*) is the death rate per day. This gives an initial size of 100 for the cohort, and the cohort population decreases according to the death rate tailored to working within two hour intervals, as each time interval represents 1/12 of a full day. As per Gething et al. it is assumed that *g*(*T*) is a function of temperature given by:

5 $$\begin{aligned} g(T)=\frac{1}{-4.4+1.31T-0.03T^2}, \end{aligned}$$

where *T* is the air temperature in degrees Celsius [15]. Based on previous studies involving *Anopholes gambiae*, this is a well-accepted function for the death rate within sensible temperature ranges [15, 29–31]. Equation (5) assumes that the death rate of mosquitoes is independent of mosquito age. This assumption is not far from reality, as mosquitoes often die from factors related to climatic variables long before age becomes a relevant factor in determining survival [29].

In Eq. (4), it was assumed that each cohort had a maximum lifespan of 33 days, or 396 time steps. This is much longer than the typical mosquito lifespans seen in the wild [32], and allows for a reduction in the amount of computation needed; even under perfect conditions, cohort population will be extremely low past 33 days. That is, in the simulations after 33 days (396 timesteps) a cohort is no longer monitored and does not contribute to the total mosquito population.

The development of sporozoites within infected mosquitoes is dependent on temperature; sporozoite development has been shown to reach completion roughly a constant number of degree days after incubation commences [15]. One degree day is one day with the temperature over the threshold by one degree. This threshold temperature varies depending on the parasite species. Here $T_{dev}$ denotes the threshold temperature required for sporozoite development to occur, and $d_{dev}$ the number of degree days required for completion of sporozoite development. With varying temperatures over time, full development of the sporozoite occurs *n* days after infection, where *n* is the minimum positive solution to:

6 $$\begin{aligned} \int _0^n H(T(\tau )-T_{dev})[T(\tau )-T_{dev}]d\tau \ge d_{dev}, \end{aligned}$$

where $H(\cdot )$ is the Heaviside function.

The focus of this study was the parasite *P. falciparum* and the mosquito species *An. gambiae sensu stricto*. This allowed setting of $T_{dev}=16$ and $d_{dev}=111$, based on previous studies [15, 29, 33, 34]. In terms of the discrete-time model, in each cohort, the sporozoite development value $Y_i$ was also tracked (Step 3, Fig. 1). Here $Y_i$ represents the number of degree days passed. Once $Y_i$ reaches a critical threshold value of $d_{dev}$, the sporozoite is considered to be fully developed. Using $T_{dev}=16$ and $d_{dev}=111$ as above, sporozoite development was tracked according to the equations:

7 $$\begin{aligned} Y_i&=0 &i\le 23,\end{aligned}$$

8 $$\begin{aligned} Y_{i+1}&=\frac{\text {max}(0,{T_i-16})}{12}+Y_i &i > 23, \end{aligned}$$

where $Y_i=0$ for $i\le 23$ in order to introduce an initial non-biting period of roughly two days shortly after the pupal stage ends. Although this non-biting period has been shown to vary with temperature, a uniform non-biting period of two days was chosen as the non-biting period does not deviate far from a length of two days across temperatures which can sustain a mosquito population [35]. During this non-biting period, no members of the population can become infected, so development of the sporozoite cannot occur. It is assumed that members of the cohort become infected immediately after this non-biting period ends.

The number of infected mosquitoes in each cohort at each timestep was then calculated (Step 4, Fig. 1). After the initial non-biting period, it was assumed that 1% of the remaining cohort would become infected [36, 37]. It is acknowledged that this is a simplification of reality and possible extensions are discussed later in the discussion.

Once sporozoite development was complete (that is, when *Y* hit the critical value of 111 degree days), the population is classified as infectious. Here, $Z_i$, represents the number of infected mosquitoes at timestep *i*, and was calculated as:

9 $$\begin{aligned} Z_i={\left\{ \begin{array}{ll} 0, \hspace{1.5cm} Y_i<111,\\ 0.01 M_i, \hspace{0.57cm} Y_i\ge 111. \end{array}\right. } \end{aligned}$$

The next step of the model (Step 5, Fig. 1) involves collating risk values across all time steps. Letting $Z_{j,i}$ be the value of $Z_i$ for the cohort starting on time step *j*, the final risk metric is then developed, $\text {risk}(t)$, at time step *t* by summing the values of $Z_i$ for all past cohorts corresponding to the time step *t*. In other words:

10 $$\begin{aligned} \text {risk}(t)=\sum ^t_{k=0} Z_{k,t-k}. \end{aligned}$$

A map was made (Step 6, Fig. 1) by visualizing the metric, $\text {risk}(t)$, at each pixel on a global scale, thus creating a spatio-temporal map of malaria risk.

### Temperature-humidity model

The temperature-based model is now extended to consider the effects of humidity. In accordance with the methods set out in Yamana and Eltahir in 2013 [30], this was done by a modification of the survival rate so that the surviving proportion over a single timestep was set to $\text {exp}(-g(T)\Delta t)S(h)$, which is dependent on both the temperature *T* and the relative humidity percentage *h*. Here $\Delta t$ is the time step of 2 h (=1/12 days). Specifically, *S*(*h*) was calculated as:

11 $$\begin{aligned} S(h)={\left\{ \begin{array}{ll} 0, \hspace{2.1cm} h \le h_{min}\\ \dfrac{h-h_{min}}{h_{max}-h_{min}}, \hspace{0.2cm} h_{min}<h<h_{max}\\ 1, \hspace{2.1cm} h \ge h_{max} \end{array}\right. } \end{aligned}$$

The factor *S*(*h*) is chosen so that humidity has no effect on survivability above an upper threshold $h_{max}$, but immediate death of the cohort occurs below a lower theshold $h_{min}$, and in between these values humidity has a linear dependence on the rate of surviving mosquitoes. Evidence from experiments on *An. gambiae* suggests that it is reasonable to set $h_{min}=5$ and $h_{max}=42$, matching empirical estimates of survival at extremely low humidities [38, 39], and at humidities more commonly experienced under field conditions [30].

### Temperature-humidity-rainfall model

The temperature-humidity model is now extended to incorporate the effect of standing water availability by altering the initial size of each new cohort. Data on the amount of standing water in a given area is not feasible to collect, considering such bodies of water can appear and disappear quickly depending on the season, and can vary in size from large lakes to small puddles. For breeding, *An. gambiae* prefer bodies of water that are temporary, contained and shallow, and which can be as small as footprints filled with water [40, 41]. It is considered that the amount of standing water in an area is linked to the amount of rainfall that area receives; this allows approximation of the transient amount of standing water without having to consider factors such as local geology. Linking the rainfall to mosquito breeding habits is a sensible assumption to make, and is supported by associations between rainfall and malaria transmission found in other studies [42].

**Fig. 2:**
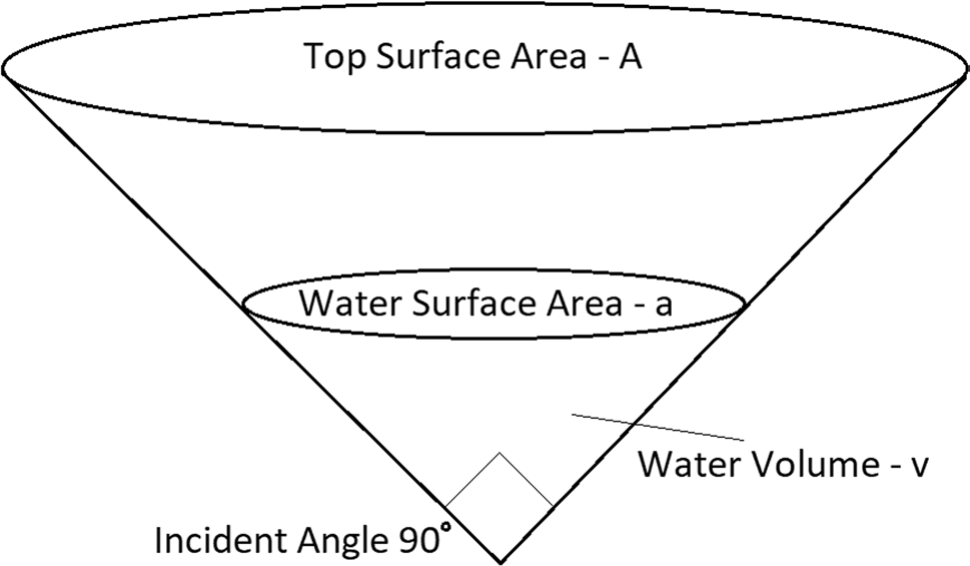
Schematic diagram of the cone used for simulating standing water

In order to simulate the amount of standing water in a given area, a variation of the method described in Parham et al. is used [43]. It is assumed that each pixel in the map contains an inverted cone with an incident angle of $90^\circ$, which holds all standing water in that region, see Fig. 2. The volume of water contained in this cone increases due to inputs from rainfall, and decreases due to loss from evaporation. Given a particular volume of water *v*, the area of water at the top of this volume can be calculated as $a=(3\sqrt{\pi }v)^{\frac{2}{3}}$. Here *a* represents the total surface area of standing water in that pixel. Note that more generally, as the slope of the inverted cone increases (e.g., incident angle decreases), the surface area of standing water available for a given volume of water would decrease.

It is assumed that each cone has a maximum capacity *V*, with a corresponding maximum surface area *A*. In order to simulate the change in water volume, the following differential equation is used:

12 $$\begin{aligned} \frac{dv}{d\tau }=pA-aE, \end{aligned}$$

where *p* is the rate of rainfall (mm per day), and *E* is the rate of water volume loss due to evaporation. This gives the rate of water input equal to the rainfall rate multiplied by the area of the opening at the top of the cone, and the rate of water output equal to the evaporation rate multiplied by the exposed surface area of the water.

The temperature and humidity model described earlier is then recomputed, but instead of setting the initial cohort size as a constant, it is set as follows:

13 $$\begin{aligned} M_0=\kappa a(\tau -\tau _d), \end{aligned}$$

which uses the water surface area *a* at time $\tau -\tau _d$. Here, $\kappa$ is a constant of proportionality and $\tau _d$ is a delay introduced in order to simulate the time between the start of the pupa stage (the last stage dependent on water) and the emergence of adult mosquitoes [23]. A linear dependence is assumed between standing water surface area and the number of mosquitoes spawned. For *An. gambiae*, $\tau _d$ was taken to be 3 days [44, 45].

### Model evaluation

Whilst it is not possible to evaluate the models of climatic suitability against equivalent empirical data, a positive correlation between the model estimate of suitability and the prevalence of malaria in Africa is expected. Therefore, the Spearman correlation coefficient is computed between the suitability estimates and malaria prevalence estimates from the Malaria Atlas Project in 2020, over 289,912 pixels in Africa within the areas for which malaria prevalence estimates are available from the Malaria Atlas Project. The choice of the Spearman rank-order correlation assumes a monotonic but not a linear relationship between the two quantities.

## Results

In total, three models were run, testing the effect of the inclusion of different bioclimatic variables, as described above. Running each of the models assigns every pixel a measure of malaria risk, $\text {risk}(t)$, for each time step, by adding up all the infected mosquitoes across all cohorts at that time. This is dependent only on temperature in the initial model ($\text {risk}_{T}$), but is dependent on temperature and humidity in the second model ($\text {risk}_{T,H}$), and is dependent on all three of temperature, humidity and rainfall in the final model ($\text {risk}_{T,H,P}$). The output of each of these models is the same resolution as the input data, with each pixel having a side length of 0.1 degrees of latitude/longitude.

**Fig. 3:**
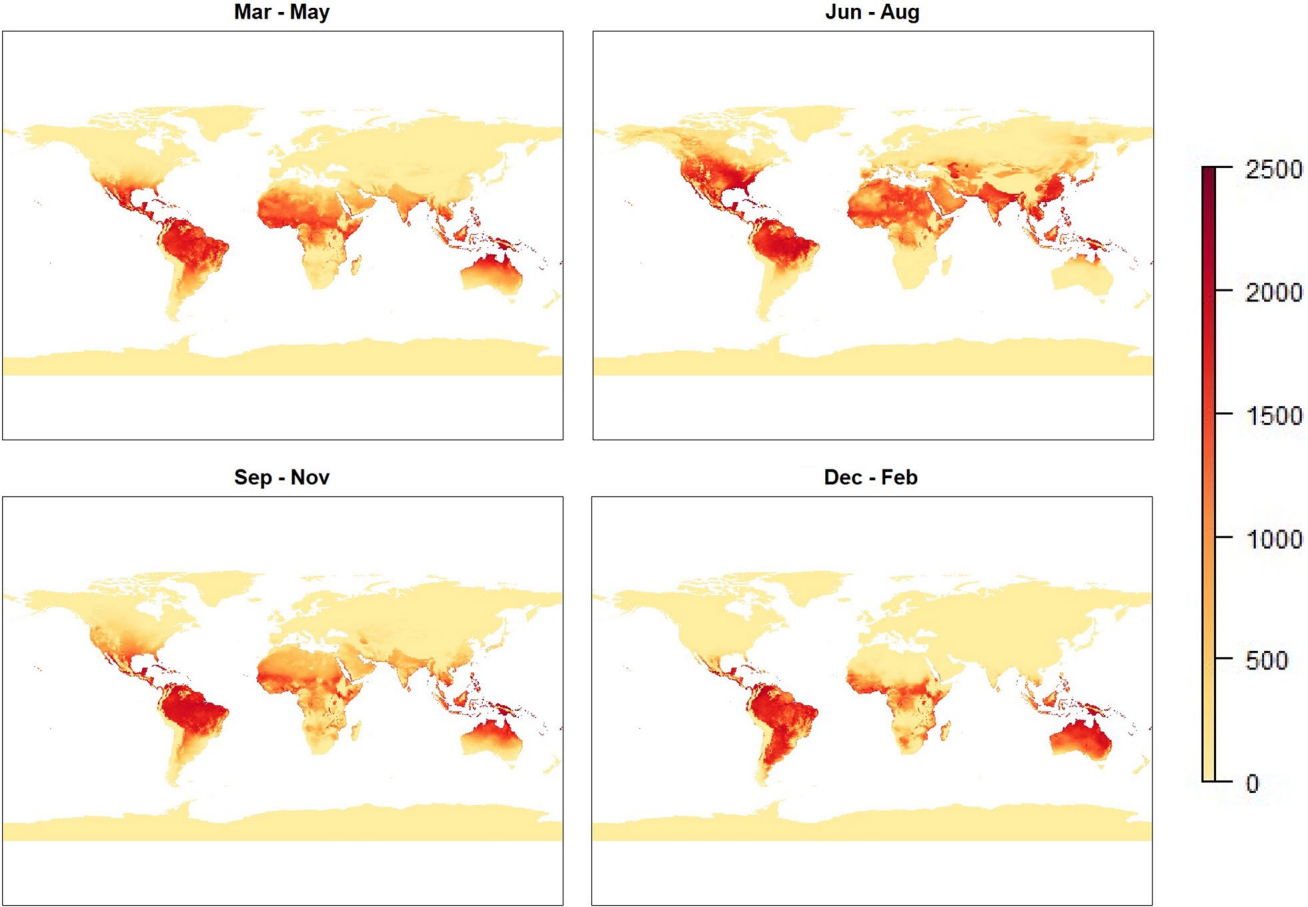
Global maps representing malaria risk based on temperature model ($\text {risk}_{T}$). Images represent seasonal averages for boreal Spring (top-left), Summer (top-right), Autumn (bottom-left) and Winter (bottom-right)

For all models the risk follows a seasonal pattern. This is most easily seen in the initial temperature based model, as this model gives a non-zero risk value to the most area, allowing how the measure of risk evolves over time to be more clearly seen. The results from this model are shown in Fig. 3.

While the results from this model highlight temperature dependence on malaria risk by displaying patterns such as seasonality, there are some obvious flaws in the results. Many arid areas, such as the Sahara or central Australia, show a significant level of risk during certain months under this model. In reality, however, it is practically impossible for mosquitoes to flourish in these environments, as mosquitoes require more humid environments with readily available water sources in order to breed and develop.

**Fig. 4:**
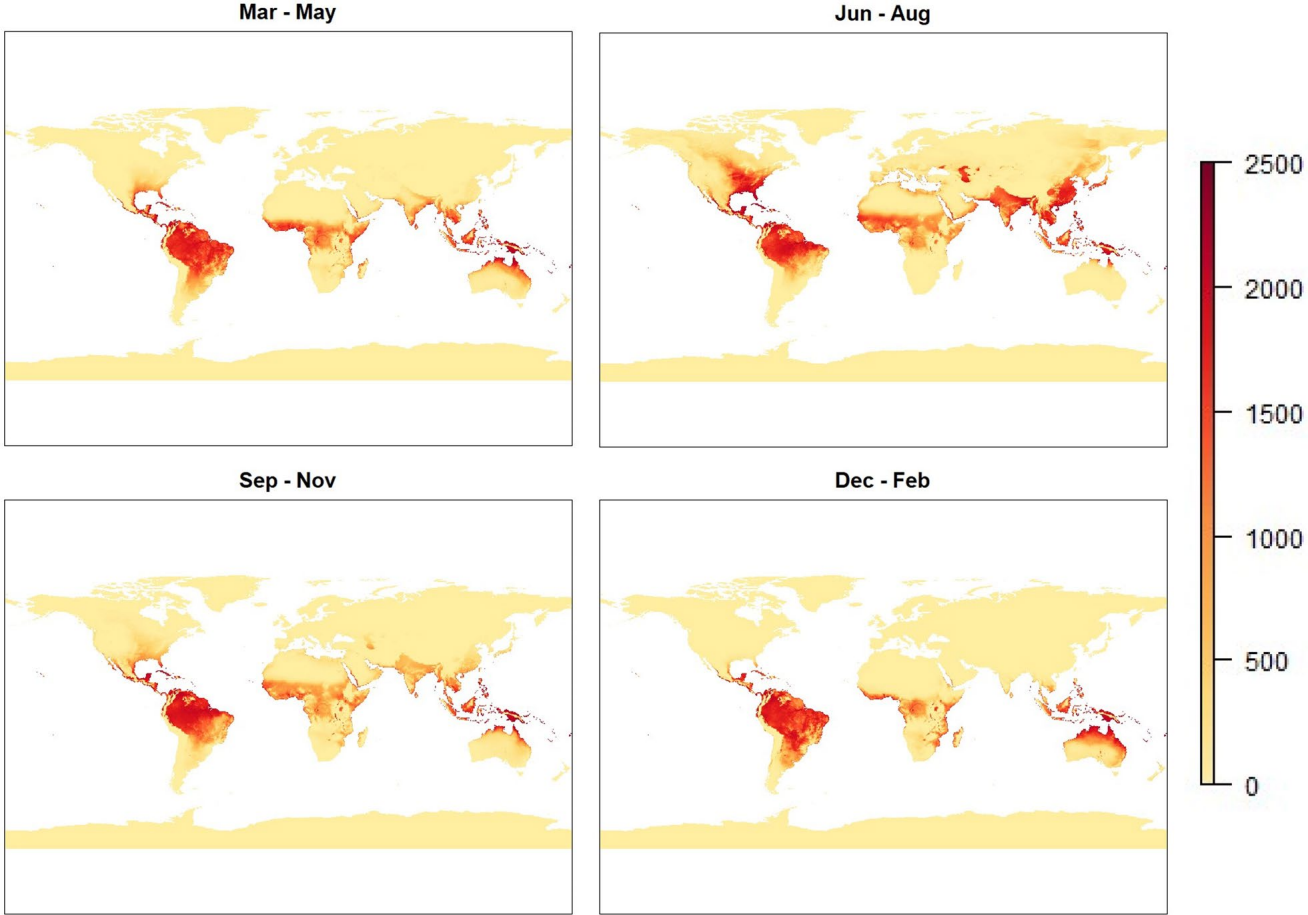
Global maps representing malaria risk, based on temperature-humidity model ($\text {risk}_{T,H}$). Images represent seasonal averages for boreal Spring (top-left), Summer (top-right), Autumn (bottom-left) and Winter (bottom-right)

Figure 4 represents results from the second model, which incorporated humidity as a factor in the calculations. From this figure, the same seasonality and general patterns from the temperature-based model can be seen at an initial glance. A closer look, however, reveals large regions in the middle of previously high-risk areas where risk drops to essentially zero. Such areas include northern Africa, central Australia and the Arabian peninsula. All these areas are generally associated with high levels of aridity and as such would be unsuitable for mosquito habitation. Note that this work has focused on creating a suitability index for malaria transmission by *An. gambiae*, not all vectors capable of *P. falciparum* transmission—there may be regions that the model has erroneously identified as suitable for malaria transmission due to this.

**Fig. 5:**
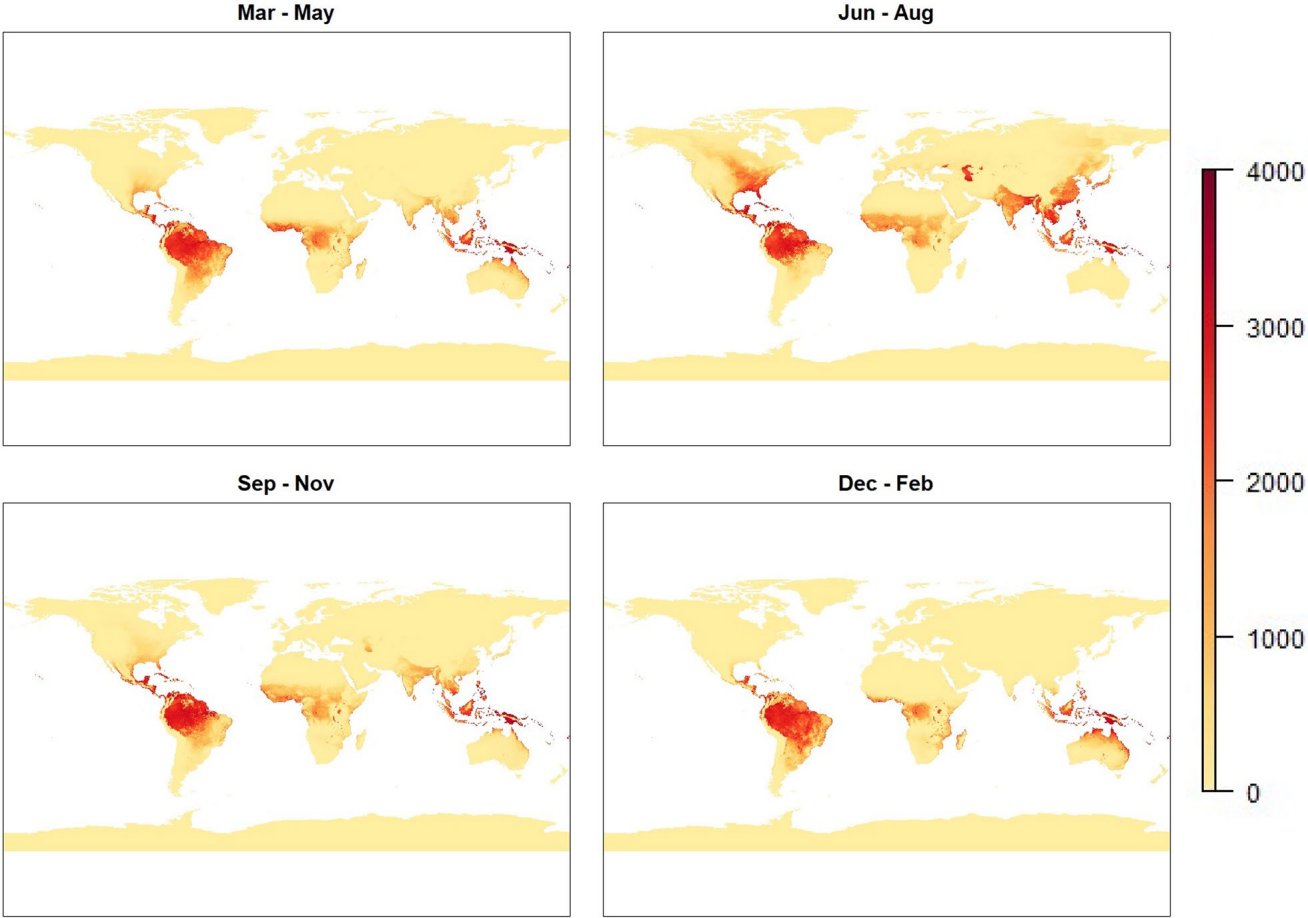
Global maps representing malaria risk, based on temperature-humidity-rainfall model ($\text {risk}_{T,H,P}$). Images represent seasonal averages for boreal Spring (top-left), Summer (top-right), Autumn (bottom-left) and Winter (bottom-right)

The third iteration of the model took rainfall into account as well. Figure 5 demonstrates the results from this iteration of the model. This allows differentiation of the risk in areas where temperature and humidity may be suitable, but where differences in rainfall patterns could lead to a relatively larger area for mosquito breeding and more suitable conditions for malaria vector development.

Using this model, the areas at relatively high risk seem to be smaller in area. Quantitatively speaking, these high-risk areas also have a higher measure of risk, as can be seen by the increased measurement scale of Fig. 5 compared to Figs. 3 and 4. This is not unexpected, since an area at the maximum water capacity produces cohorts with higher initial numbers than in the previous two models.

In this version of the model, again low risk measurements in arid areas can be seen. There is also a higher weighting of risk in areas that are associated with tropical rainforest, such as Indonesia, the Amazon Basin, the Congo Basin and the coast of Western Africa. Areas typically associated with grasslands, such as Northern Africa, Eastern Africa and South Brazil, still show some level of risk, but this level is reduced relative to the model’s hotspots. This is especially noticeable in the months where these regions displayed the highest risk when using the previous model (boreal summer for Northern Africa, boreal winter for Eastern Africa and South Brazil).

**Fig. 6:**
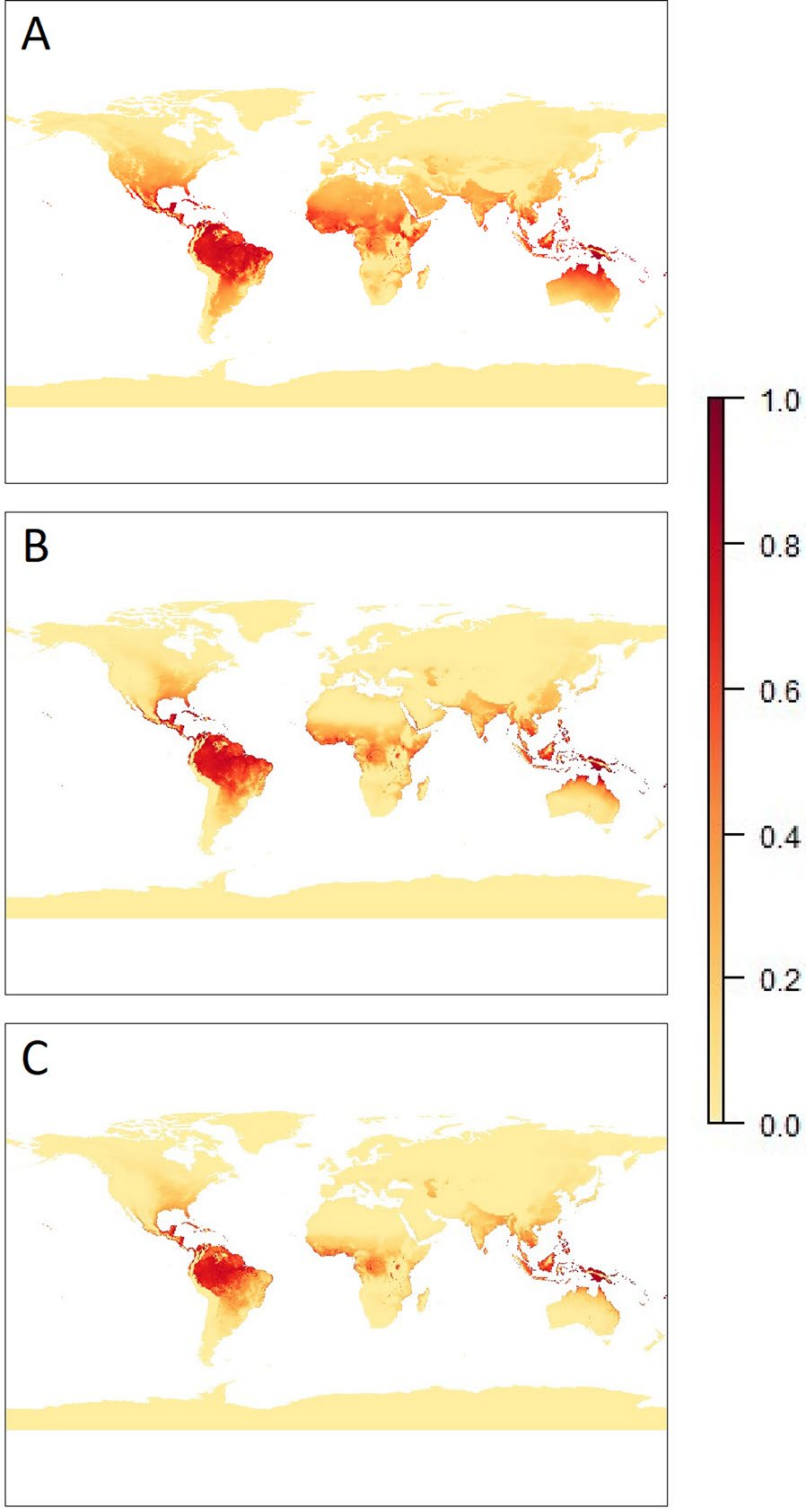
Comparison of the scaled average results across all months of (**A**) the temperature-based model ($\frac{\text {risk}_T}{\text {max(risk}_T)}$) (**B**) the model incorporating temperature and humidity ($\frac{\text {risk}_{T,H}}{\text {max(risk}_{T,H})}$) (C) the model incorporating temperature, humidity and rainfall ($\frac{\text {risk}_{T,H,P}}{\text {max(risk}_{T,H,P})}$). For these models, $\text {max(risk}_T)$ and $\text {max(risk}_{T,H})$ both evaluate to approximately 2336.5, and $\text {max(risk}_{T,H,P})$ evaluates to approximately 3703.9

The increase in relative risk of more moist areas such as rainforest can be seen more clearly in Fig. 6. This shows a comparison of the average results across all months for the three models and indicates the restriction placed on suitability by adding humidity and rainfall to the model, respectively.

There is weak agreement between the Malaria Atlas Project median estimates of *P. falciparum* prevalence in Africa and the estimates of suitability solely based on temperature (Spearman Correlation coefficient of $\rho = 0.24$). The addition of humidity and then rainfall improves the comparison ($\rho = 0.62$ when humidity added; $\rho =0.70$ when both humidity and rainfall added). Of course, there will be regions where *P. falciparum* prevalence is low where the models predict that the environment is suitable for transmission. This is most notable for the temperature-only model where large parts of the Sahara desert are predicted to be suitable for malaria since the effect of low humidity (and low rainfall) are not taken into consideration.

## Discussion

In this paper, a suitability index for the transmission of *P. falciparum* that varies with temperature, humidity and rainfall has been developed. The equations that characterize the relationships have been established previously in the literature, but this is the first model to incorporate all three factors into a single mathematical model. Global malaria suitability maps based on temperature only, temperature and humidity, and temperature, humidity and rainfall have been presented.

There are several limitations of this work that should be acknowledged. Firstly, mosquitoes adapt to their environment to take advantage of human housing for their own comfort. In this way, the temperature and humidity that mosquitoes experience depends on the living conditions of humans in the area. Secondly, a suitability index for *An. gambiae* on a global scale has been considered here, however this species largely only exists in Africa. Hence, for regions outside Africa, the results presented here should be interpreted loosely to reflect the suitability for other *Anopheles* species (albeit with a different parameterization). In future, parallel models for major vector species could be developed; for example *Anopheles funestus* and *Anopheles coluzzii* in Africa and *Anopheles stephensi* in India (now also present in Africa). Thirdly, the notion that 1% of mosquitoes per time-step are infected is arbitrary and another approach could be to, for example, assume that by day 3 all mosquitoes have been infected.

A further improvement on the work presented would be to incorporate future environmental predictions so that trends in time can be assessed, including the effects of climate change. This would also allow the effect of climatic factors such as El Niño events that affect precipitation volumes to be assessed. Improvements to the humidity model could include modelling the nonlinear effects between the extreme humidity levels (see Eq. 11) while the standing surface area water model could be improved by considering location-dependent ‘flooding’. In the model implementation, the number of mosquitoes born at each timestep was either constant or linearly dependent on surface water available. It would be interesting to investigate the role of a spatially-varying carrying capacity that controls the generational dependence of clutch size. Likewise, the model would be improved by incorporating contemporary, dynamic temperature data to better characterize its multifaceted relationship with vector ecology and the sporogonic cycle. Such an approach was taken by Weiss et al. [16], who utilized dynamic air temperature estimates that were modelled from satellite-imagery-derived land surface temperature.

The model framework presented in this paper is highly flexible and can be readily coupled to other more detailed models, such as mosquito population dynamic models and malaria transmission models. While this would require thought on the nature of coupling of the models, it would no doubt provide interesting insight into malaria at the human population level. The suitability maps that have been generated here are also potentially important covariates for use in spatio-temporal mapping of malaria prevalence at global and national levels. These prevalence maps, and by extension the ones generated here, are particularly important for countries that are targeting malaria control and elimination, in that they can guide the allocation of scarce resources to where they are most needed.

## Data Availability

No datasets were generated or analysed during the current study.
